# Providing height to pullets does not influence hippocampal dendritic morphology or brain-derived neurotrophic factor at the end of the rearing period

**DOI:** 10.1016/j.psj.2022.102161

**Published:** 2022-08-30

**Authors:** Allison N. Pullin, Victoria S. Farrar, Jason W. Loxterkamp, Claire T. Jones, Rebecca M. Calisi, Kristina Horback, Pamela J. Lein, Maja M. Makagon

**Affiliations:** ⁎ Center for Animal Welfare, Department of Animal Science, University of California, Davis, CA 95616, USA; † Animal Behavior Graduate Group, College of Biological Sciences, University of California, Davis, CA 95616, USA; ‡ Department of Neurobiology, Physiology and Behavior, University of California, Davis, CA 95616, USA; § Department of Molecular Biosciences, University of California, Davis, School of Veterinary Medicine, Davis, CA 95616, USA

**Keywords:** brain-derived neurotrophic factor, chicken, Golgi stain, hippocampus, rearing environment

## Abstract

Pullets reared with diverse behavioral experiences are faster to learn spatial cognition tasks and acclimate more successfully to laying environments with elevated structures. However, the neural underpinnings of the improved spatial abilities are unclear. The objective of this study was to determine whether providing structural height in the rearing environment affected the development of the hippocampus and whether hippocampal neural metrics correlated with individual behavior on spatial cognition tasks. Female Dekalb White pullets were reared in a floor pen (**FL**), single-tiered aviary (**ST**), or two-tiered aviary (**TT**; 5 pens/treatment). Pullets completed floor-based Y-maze and elevated visual cliff tasks to evaluate depth perception at 15 and 16 wk, respectively. At 16 wk, brains were removed for Golgi-Cox staining (n = 12 for FL, 13 for ST, 13 total pullets for TT; 2 to 3 pullets/pen) and qPCR to measure gene expression of brain-derived neurotrophic factor (*BDNF*; n = 10 for FL, 11 for ST, and 9 pullets for TT). Rearing environment did not affect various morphometric outcomes of dendritic arborization, including Sholl profiles; mean dendritic length; sum dendritic length; number of dendrites, terminal tips, or nodes; soma size; or *BDNF* mRNA expression (*P* > 0.05). Hippocampal subregion did affect dendritic morphology, with multipolar neurons from the ventral subregion differing in several characteristics from multipolar neurons in the dorsomedial or dorsolateral subregions (*P* < 0.05). Neural metrics did not correlate with individual differences in behavior during the spatial cognition tasks. Overall, providing height during rearing did not affect dendritic morphology or *BDNF* at 16 wk of age, but other metrics in the hippocampus or other brain regions warrant further investigation. Additionally, other structural or social components or the role of animal personality are areas of future interest for how rearing environments influence pullet behavior.

## INTRODUCTION

Pullets (*Gallus gallus domesticus*) grow and develop in rearing environments until just prior to the onset of egg laying, approximately 16 to 18 wk of age, at which time they are transitioned into their laying environments. During this early period of life, pullets are sensitive to environmental factors that influence cognitive, behavioral, and physical development (reviewed in Campbell et al., 2019). Multiple studies have linked rearing environments to pullet and hen spatial abilities. Pullets reared with elevated structures, access to outdoor range, or novel sensory enrichment were faster to learn spatial cognition tasks (i.e., Y-maze, holeboard task, T-maze, and detour task; Krause et al., 2006; Tahamtani et al., 2015; Campbell et al., 2018; Norman et al., 2019) than pullets reared without these environmental components. Pullets reared with access to elevated space also acclimated faster to novel environments as they used elevated structures more and had fewer collisions compared to pullets reared on the floor or in conventional cages (Gunnarsson et al., 2000; Brantsæter et al., 2016; Pullin et al., 2020). While it is not possible to disentangle whether the early-life experiences influenced cognition or physical ability, these findings suggest that environments offering more diverse behavioral experiences during rearing modify pullet development such that they can more effectively process spatial information. However, the neural basis of these developmental changes has yet to be described.

The hippocampus brain region is an area of interest for studying the neural underpinnings of spatial abilities in chickens, as it is associated with spatial orientation, navigation, and memory in avian species (Krebs et al., 1989; Bingman and Mench, 1990; Tommasi et al., 2003). Previous research failed to find a link between pullet rearing environment and hippocampal volume or tyrosine hydroxylase, an enzyme involved in dopamine synthesis (Tahamtani et al., 2016; Campbell et al., 2018). However, hippocampal volume is a coarse metric of brain function, whereas finer measurements of neuronal cytoarchitecture can offer biologically relevant insight into the cellular and synaptic mechanisms underlying spatial cognition (Roth et al., 2010). Further investigation into neural correlates of plasticity is, therefore, warranted.

Brain function is determined in large part by the pattern of synaptic connections, which is strongly influenced by experience-dependent changes in the complexity of dendritic branching (Nithianantharajah and Hannan, 2006). Golgi staining is a technique used to visualize and quantify the dendritic arbors of individual neurons in the intact brain by stochastically filling intact cell bodies and their axonal and dendritic processes (Carter and Shieh, 2015). In studies modeling experience-induced plasticity in mammals, the technique has been used to evaluate the effects of environmental enrichment on neuronal cytoarchitecture. Several studies have reported increased hippocampal dendritic arborization as a result of environmental enrichment (Berman et al., 1996; Faherty et al., 2003; Darmopil et al., 2009; Nithianantharajah et al., 2009). However, we are aware of only one study using Golgi staining to assess experience-induced plasticity in the chicks’ hippocampal cytoarchitecture (Freire and Cheng, 2004). Chicks reared with visual barriers to their imprinting stimulus experienced changes in perception (visual occlusion) and physical access to the stimulus. Those chicks had longer dendrites and more dendritic spines in the hippocampus at 16 d of age than chicks reared without visual barriers, suggesting that the former had an advantage at processing spatial information. The authors concluded that these changes were hippocampal-specific, after finding no changes in the nidopallium (formerly neostriatum; Reiner et al., 2004) of the brain. Within the hippocampus, the right hemisphere had a higher degree of dendritic development than the left hemisphere, suggesting that the right hemisphere was more involved in spatial processing than the left (Freire and Cheng, 2004). Therefore, the right hemisphere hippocampus is an ideal candidate for investigating how pullet rearing environments modify neuronal cytoarchitecture and consequently birds’ abilities to process spatial information.

Brain function is also influenced by the prevalence of certain molecules that are involved in regulating neuronal plasticity. Brain-derived neurotrophic factor (***BDNF***) is a growth factor implicated in activity-dependent plasticity, learning, and memory due to its roles in cell survival, cell proliferation, and synaptic transmission regulation (Miranda et al., 2019). The highest levels of *BDNF* gene expression were found in the hippocampus of adult mice (Hofer et al., 1990). In mammals, higher expression of hippocampal *BDNF* has been linked with environmental enrichment that promotes exercise, socialization, novelty, or a combination (reviewed in van Praag et al., 2000; Rossi et al., 2006; Cao et al., 2017). We are aware of only one study measuring the expression of *BDNF* as an outcome of providing pullets with different rearing environments. Pullets reared with perches were less fearful, had lower levels of plasma corticosterone, and higher levels of *BDNF* gene expression in the hypothalamus compared to pullets reared without elevated structures, suggesting that rearing environment influences neural correlates for stress mediation in the hypothalamus (Yan et al., 2020). However, investigating the influence of the rearing environment on hippocampal *BDNF* as a metric of spatial cognition is understudied in pullets.

If neuronal cytoarchitecture and gene expression of *BDNF* are successful neural metrics for chicken spatial cognition, then correlating these metrics to individual pullet behavior on spatial cognition tasks could provide more insight into neural underpinnings of individual differences in behavior. In humans, brain image analyses revealed that activity in the hippocampus correlated with individual differences in spatial learning strategies (Shelton and Gabrieli, 2004), and hippocampal volume correlated with individual differences in sense-of-direction (Burte et al., 2018). In adult hens, individual differences in hippocampal cell proliferation correlated with the amount of time that individual hens utilized an outdoor range (Armstrong et al., 2020). The relationship between brain function and performance on spatial cognition tasks has yet to be described in pullets though. Depth perception is an aspect of spatial cognition that can be measured utilizing tasks such as a visual cliff (Shinkman, 1962; Jones, 2021) or Y-maze with asymmetrical arms (Jones, 2021). The visual cliff task evaluates the birds’ willingness to move across a perceived vertical gap set to various depths, whereas the Y-maze requires the birds to discriminate depth in the horizontal plane. Individual birds differ in their strategies to complete these tasks. Individual differences in the number of times pullets look down at the edge of the visual cliff to gather information prior to crossing, latency to cross the visual cliff, latency to exit the Y-maze, and choice of Y-maze exit arm have been reported (Jones, 2021). These tasks require pullets to estimate depth and distance, which is commonly associated with visual pathways that link multiple visual cognition regions of the avian brain (including the nidopallium caudolaterale region, NCL; Niu et al., 2022). Recent research found that the hippocampus is also involved in avian visual cognition through its connection to the NCL (Niu et al., 2022). The hippocampus forms memories from visual perception of the environment that can be retrieved later to distinguish spatial stimuli and determine distances in spatial tasks (Lee et al., 2012; Theves et al., 2019; Yang and Naya, 2020; Treder et al., 2021). Therefore, hippocampal development may aid pullets in completing these tasks, particularly if they have previous experience and memory formation with varying distances and depths in their rearing environments.

The primary aim of this study was to determine whether providing height in the rearing environment affected the development of the hippocampus, specifically regarding neuronal cytoarchitecture and *BDNF* expression. We predicted that pullets reared with the most height would have a higher degree of dendritic arborization in hippocampal multipolar neurons as indicated by Sholl profiles with more intersections at any given distance from the soma, higher values for non-Sholl dendritic metrics, and higher gene expression of hippocampal *BDNF* compared to pullets reared with intermediate or no height. This study also aimed to determine whether hippocampal neural metrics correlated with individual behavior in spatial cognition tasks. We hypothesized that hippocampal metrics would explain individual differences in behavior in Y-maze and visual cliff spatial cognition tasks. We predicted that some or all of the non-Sholl metrics and gene expression of *BDNF* would correlate with the number of times looking down, crossing, and latency to cross a visual cliff at 30 and 90 cm heights, as well as choosing the short arm and latency to exit the arena in a Y-maze task.

## MATERIALS AND METHODS

All experimental procedures were reviewed and approved by the University of California, Davis Institutional Animal Care and Use Committee (Protocol #20307).

### Pullet Housing and Management

**Figure 1:**
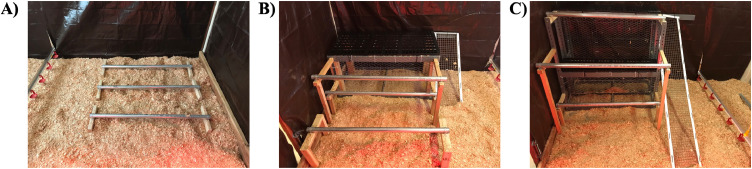
Female Dekalb White pullets were reared in one of three environments for the first 16 wk of life: floor rearing environment or FL (A), a single-tiered aviary or ST (B), and two-tiered aviary or TT (C).

Dekalb White pullets (N = 835) were raised in 15 pens (3.05 × 3.05 × 2.74 m, L × W × H) in one building at the Hopkins Avian Facility at University of California, Davis (Davis, CA). The pullets were obtained at 1 d of age from a commercial hatchery and randomly assigned to one of 3 rearing environments (55–56 pullets/pen, 5 pens/rearing environment): floor (**FL**), single-tiered aviary (**ST**), or two-tiered aviary (**TT**; Figure 1). The building was divided into five blocks of 3 pens/block to ensure that rearing environments were distributed throughout the building. Rearing environments were randomly assigned to pens within each block. At 8 wk of age, a portion of pullets were removed from each pen as part of data collection procedures for another component of this project, resulting in a density of 45 pullets/pen until data collection at 16 wk.

From 1 d of age, pullets were bedded with pine wood shavings (Mallard Creek Inc., Rocklin, CA). Water was provided ad libitum via automatic water lines (Lubing USA, Cleveland, TN) with 12 nipples /pen. A start and grow diet (Purina Start and Grow Medicated Crumbles, Purina Animal Nutrition LLC, Gray Summit, MO) was provided ad libitum using two 13.6 kg round feeders/pen (52 cm circumference/feeder). Temperature and artificial lighting were maintained according to the Dekalb White Product Guide (Dekalb, the Netherlands). Husbandry personnel entered each pen one time daily to monitor and make adjustments for feed, water, litter, lights, ventilation, and animal health.

In FL pens, pullets had access to four metal floor perches (121.9 L, 3.8 cm diameter) at 10.5 cm from the floor. In ST pens, pullets could access an aviary structure containing 3 elevated metal perches (121.9 L, 3.8 cm diameter) installed at the heights of 35.4 cm (2 perches) and 64.7 cm from the floor (third perch), as well as one elevated tier with a plastic slatted surface (121.9 × 61.0 cm, L × W; Dura-Slat Poultry and Kennel Flooring, Southwest Agri-Plastics, Inc., Addison, TX) located 62.9 cm off the floor. A wire mesh ramp was provided to facilitate access to the tier (96.5 × 31.8 cm, 40-degree angle; McNichols Wire Mesh, McNichols Co., Inc., Livermore, CA). A single metal floor perch, identical to the ones used in FL pens, was placed adjacent to the structure. The aviary structure in the TT pens contained 3 elevated metal perches (121.9 L, 3.8 cm diameter) located 29.4, 89.9, and 125.7 cm from the floor, as well as 2 elevated tiers with plastic slatted surfaces (121.9 × 30.5 cm, L × W) located 62.9 cm and 123.8 cm from the floor. A wire mesh ramp facilitated access to both tiers (190.5 × 31.8 cm, 40-degree angle). A metal floor perch was provided next to the aviary structure. In ST and TT, chicken wire was used to prevent pullets from accessing floor space directly underneath the tiers of the aviary in order to keep stocking density constant across all 3 rearing environments.

To ensure that birds’ visual experiences were limited to their own rearing environments, heavy-duty, plastic tarps (3.05 × 1.83 m, L × W; Everbilt, The Home Depot, Inc., Atlanta, GA) were hung on pen walls and doors to reduce visibility into adjacent pens and the hallway used by human caretakers and researchers. The top half of the pen (0.91 m between where the tarp ended and the ceiling) was not covered so that ventilation through the building was not obstructed. As a result, TT birds standing on the highest tier could see over the tarp at approximately 8 wk of age, gaining additional visual cues that pullets in ST and FL rearing environments did not have.

### Individual Behavior During Spatial Cognition Tasks

At 15 wk of age, 2 to 3 pullets were randomly selected from each pen, received a leg band for individual identification, and tested individually in a Y-maze (n = 14 pullets/rearing environment; N = 42 pullets total). At 16 wk of age, the same pullets were tested individually on a visual cliff. Testing at each age occurred across 5 d, where one pen from each rearing environment was tested/d (3 pens total tested/d). The order of rearing environment tested was randomized daily to prevent order bias. Construction of the tasks, procedure used during the testing, as well as methods for behavioral observations are described in detail in Jones (2021). In brief, the Y-maze task required individual birds to choose one arm of the maze to exit into an arena in 2 consecutive trials. In one trial, the Y-maze arms were equal length (1:1 ratio; 90 cm each), and in another trial the arms were unequal length (1:3 ratio; 30 and 90 cm). The order of trials was randomized across birds to prevent order bias, and the side of the longer arm (right or left) was also randomized across trials to prevent side bias. Trials were considered complete after the bird had exited one of the arms or after 150 s had elapsed, whichever came first. For the scope of the present study, we aimed to correlate neural metrics with the most spatially challenging tasks; therefore, we evaluated individual pullet behavior only during the unequal length trial. We recorded which arm the pullet exited from during the unequal length trial (short or long) as well as the latency to complete the trial.

The visual cliff task required individual birds to cross over a table that was fitted with checkerboard material and topped with plexiglass, a design that created illusions of depth without risk of the bird falling (e.g., Shinkman, 1962). Pullets were tested in 3 trials of different heights in a randomized order: 15, 30, and 90 cm, where the latter 2 heights created perceived depth. Trials were considered complete after the bird had crossed over the cliff to a platform at the end of the table or after 90 s had elapsed, whichever came first. Similar to the Y-maze, we aimed to correlate neural metrics with the most spatially challenging tasks; therefore, we evaluated individual pullet behavior only during the 30 and 90 cm height trials. We recorded the number of times the bird looked down during the trial, whether or not the pullet crossed the table, and the latency to complete the trial.

### Brain Tissue Collection

Immediately after completing the visual cliff task at 16 wk of age, each pullet was euthanized using an overdose of isoflurane inhalant, followed by rapid decapitation for brain tissue collection (n = 14 pullets/rearing environment). Brains were removed in less than three minutes from decapitation and hemisected. The right hemisphere was immediately placed into impregnation solution for Golgi-Cox methods described below. The left hemisphere was flash frozen on dry ice and stored in a −80°C freezer until quantitative polymerase chain reaction (**qPCR**) analysis described below.

### Golgi-Cox Staining

Golgi-Cox staining was performed on the right hemisphere tissue using the FD Rapid GolgiStain Kit (FD NeuroTechnologies, Inc., Columbia, MD) following modified manufacturer's instructions and as previously described (Lein et al., 2007; Keil et al., 2017). During impregnation in Solutions A and B, instructions were modified for pullets by using an estimated hemispheric brain volume of 2 cm^3^ (estimate based on whole chicken brain volumes that were previously reported as 3.3 to 3.8 cm^3^ for Brown Leghorn, Frahm and Rehkämper, 1998, and 4.3 cm^3^ for *Gallus gallus domesticus* broadly, Kawabe et al., 2009). Further modifications were made after immersion in Solution C, where tissue was submerged in 10% sucrose in PBS for 4 h at 4°C, then stored in 30% sucrose in PBS at 4°C for approximately 7 mo until sectioning. Prior to sectioning, the right hemisphere tissue was transferred to 70% ethanol in distilled water for 15 min at 4°C, then sectioned coronally at 100 μm on a vibratome set to a frequency of 70 Hz and speed of 0.40 mm/s (VT-1000, Leica Biosystems Inc., Buffalo Grove, IL). Sections (interaural 0.40–3.76 mm, Puelles et al., 2007) were mounted on gelatin-subbed slides and dried for a maximum of 2 wk at room temperature in the dark. The final stain was applied to sections with Solutions D and E following the manufacturer's instructions, then sections were dehydrated and topped with a coverslip using Permount Mounting Medium (Thermo Fisher Scientific, Waltham, MA). A portion of pullets were excluded from further analysis due to errors that occurred during sectioning or the final stain steps that made imaging neurons in the hippocampus impossible (n = 2 pullets for FL, 1 pullet for ST, and 1 pullet for TT).

**Figure 2:**
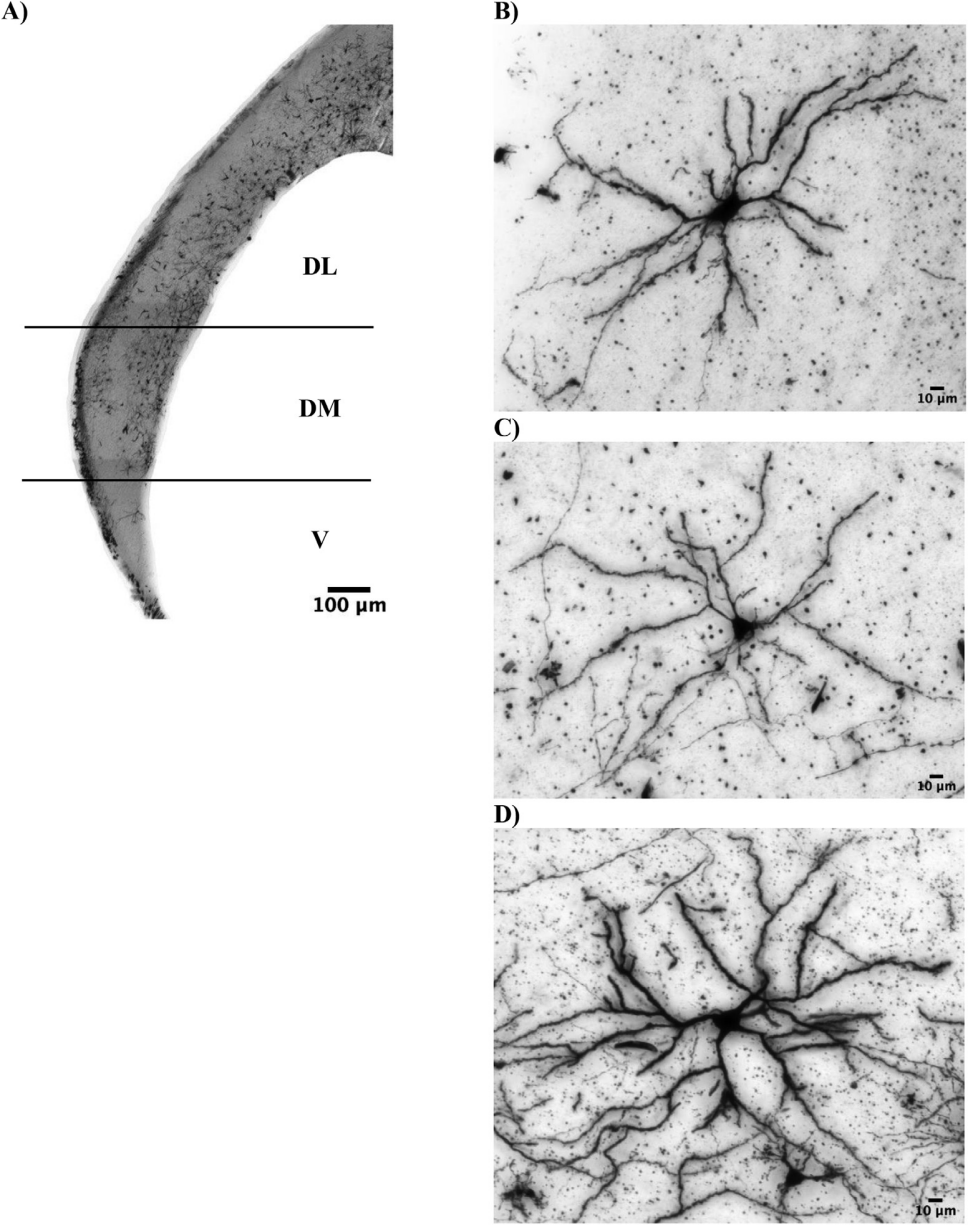
A pullet hippocampus was imaged with three images at 4X, then fused together with ImageJ software to create a representative image of the brain region (A). Subregions of the hippocampus were distinguished for ventral, V, dorsomedial, DM, and dorsolateral, DL for neuron imaging. Neurons were imaged at 20X for female Dekalb White pullets at 16 wk of age that were raised in one of three rearing environments. Representative photomicrographs of Golgi-stained hippocampal multipolar neurons at 20X magnification from the floor rearing environment or FL (B), a single-tiered aviary or ST (C), and two-tiered aviary or TT (D).

Brightfield image stacks of hippocampal multipolar neurons were taken at 20× magnification in 0.2 μm steps using an IX-81 inverted microscope (Olympus, Shinjuku, Japan) and MetaMorph Image Analysis Software (version 7.1, Molecular Devices, Sunnyvale, CA). One to 5 neurons per animal were imaged from the hippocampal coronal slices of individual pullets, ranging from interaural 1.12 to 3.76 mm (n = 12 pullets for FL, 51 total neurons; n = 13 pullets for ST, 54 total neurons; n = 13 pullets for TT, 52 total neurons; see Figure 2 for representative images). Criteria for consistent and accurate selection of neurons included 3 conditions previously described (Lein et al., 2007): 1) well-impregnated neurons with no evidence of incomplete or artificial staining, 2) blood vessels, glia, or non-descript precipitate do not obscure neuron or branches, and 3) the cell body is located in the middle third of the thickness of the section. Additional criteria were incorporated for pullets, including 4) neurons are multipolar projection neurons and have at least four thick spinous dendrites coming out around an ovoid or spherical-shaped soma (Tömböl et al., 2000) and 5) hippocampal subregion will be identified for the multipolar neuron at time of imaging (Figure 2). The ventral subregion (**V**) is defined as the V-shaped region at the caudal end of the hippocampus. Once the hippocampus begins to widen, the dorsomedial region (**DM**) begins. This region widens and then narrows again. Once the region narrows into a consistent width for the remainder of the hippocampus, it is the dorsolateral region (**DL**). These distinctions align with previous work evaluating avian hippocampal subregions (Atoji and Wild, 2004; Smulders, 2017). One pullet may have neurons imaged from more than one subregion, so the final sample size based on subregion was n = 36 pullets for the largest region DL, 109 total neurons; n = 20 pullets for DM, 35 total neurons; n = 9 pullets for V, 13 total neurons (see Table 1 for the number of neurons imaged/subregion/pullet). At least 2 neurons from each rearing environment were represented in each subregion.

**Table 1:** Number of multipolar projection neurons collected for Golgi-Cox analysis from each hippocampal subregion for individual pullets.

Rearing environment and animal ID	Hippocampal subregion neurons, n			Total neurons, n
FL	V	DM	DL	
3.12	1	1	3	5
3.13	3	0	1	4
3.16	1	1	3	5
6.26	0	1	4	5
8.31	0	1	3	4
8.39	0	2	3	5
8.40	0	0	2	2
16.73	0	0	5	5
16.79	0	0	5	5
19.24	2	0	3	5
19.28	0	0	1	1
19.29	0	0	5	5
ST				
1.38	0	0	5	5
1.39	0	3	2	5
1.40	0	2	3	5
5.84	1	2	2	5
5.88	0	0	3	3
13.47	0	0	3	3
13.48	0	2	3	5
13.50	0	1	3	4
14.3	0	0	5	5
14.7	0	1	2	3
14.9	0	0	2	2
18.91	1	2	1	4
18.92	0	0	3	3
18.95	0	1	4	5
TT				
2.44	1	0	4	5
2.45	0	3	2	5
2.48	2	1	2	5
4.62	0	3	2	5
4.68	0	1	1	2
7.54	0	5	0	5
7.56	0	1	4	5
7.58	0	0	2	2
15.11	0	1	4	5
15.20	0	0	5	5
17.3	0	0	5	5
17.7	0	0	1	1
17.8	1	1	0	2

Female Dekalb White pullets were reared in one of three environments until 16 wk: floor pens (FL), single-tiered aviary (ST), or two-tiered aviary (TT).

Hippocampal multipolar projection neurons were imaged from the ventral (V), dorsomedial (DM), and dorsolateral (DL) subregions.

Dendrites and somas from all imaged neurons were manually traced by one experimenter using NeuroLucida (version 11, MBF Bioscience, Williston, VT). Arbor complexity was quantified by automated Sholl analysis (NeuroLucida Explorer, version 11, MBF Bioscience), where the sum of intersections are reported between neuronal dendrites and 10-μm Sholl rings centered on the neuronal soma. Area under the curve (**AUC**, 0–260 μm) was calculated for the Sholl curve using Prism (version 9, GraphPad Software, San Diego, CA), including comparisons between proximal (0–40 μm) versus distal AUC (40–260 μm). The 40 μm Sholl ring was used to separate proximal versus distal because it was the peak for all three Sholl curves for the rearing environments. Non-Sholl neuronal metrics included mean dendritic length, sum dendritic length, number of dendrites, number of terminal tips, number of nodes, and soma size. The experimenter was not completely blind to rearing environment during staining, imaging, or tracing because pen numbers were associated with sample identification. However, the experimenter was not able to readily identify the rearing environment associated with pen numbers for the majority of the pens.

### qPCR Analysis

The left hemisphere was sectioned coronally at 100 μm on a cryostat (CM 1860, Leica Biosystems Inc.) for microdissection of the hippocampus using a 3 mm punch (an established approach used for gene expression analysis in avian neural tissue; MacManes et al., 2017; Bauer et al., 2018; Lattin et al., 2022). Circular punches were taken from the most superior/cranial, medial portion of the sectioned hemisphere above the lateral ventricle, following the chick brain atlas (interaural −0.08 to 3.76 mm, Puelles et al., 2007; see Figure 5A for a representative punch). As we aimed to capture as much of the hippocampus as possible with this approach, punches may also include some residual tissue from the medial and lateral pallium. Punched tissue (mean ± SD punch weight: 19.8 ± 5.2 mg) was stored in a −80°C freezer until RNA extraction. To extract total RNA and run real-time qPCR reactions, we followed the methods previously described for avian brain nuclei tissue in Farrar et al., (2022). Briefly, RNA was extracted using a modified Direct-zol RNA Miniprep kit (Zymo Research, Irvine, CA) and RNA quality was evaluated using a Nanodrop ND-1000 spectrophotometer (Thermo Fisher Scientific, Wilmington, DE). Samples were excluded from analysis if their 260/280 and 260/230 ratios were <1.8 or if their RNA concentration was low (<100 ng/µL), which resulted in the exclusion of n = 4 pullets for FL, 3 pullets for ST, and 5 pullets for TT. The final sample size for qPCR was n = 10 for FL, n = 11 for ST, and n = 9 pullets for TT. RNA was then treated with DNase I (Invitrogen, Waltham, MA) to remove any remaining genomic DNA, and converted to complementary DNA (**cDNA**) using qScript cDNA Supermix (Quantabio, Beverly, MA). cDNA was diluted 5-fold prior to qPCR analysis. qPCR for each sample was run in triplicate on a CFX384 Touch Real-time PCR detection system (Bio-Rad Laboratories, Hercules, CA) using the reaction mix and cycling protocol described in Farrar et al., (2022). All samples were run on a single qPCR plate, including negative controls (i.e., H_2_O) to verify that there was no contamination. Similar to the Golgi-Cox methods, the experimenter was not completely blind to rearing environment during all stages for qPCR analysis due to sample identification being associated with the pen number.

We ran qPCR for the gene of interest, brain-derived neurotrophic factor (***BDNF***), in addition to 2 reference genes, beta-actin (***ACTB***) and peptidylprolyl isomerase A (***PPIA***). These reference genes have previously been shown to be stable in avian neural tissue (Zinzow-Kramer et al., 2014). We verified that rearing environment did not have a significant effect on mean reference gene expression for *ACTB* (*F*_2,11_ = 0.44, *P* = 0.66) or *PPIA* (*F*_2,11_ = 1.33, *P* = 0.31). Primers used for qPCR were designed on species-specific gene sequences for *Gallus gallus* using the NCBI Primer-BLAST tool (Table 2). Primers were then validated by running a 10-fold serial dilution to determine amplification efficiencies (mean ± SD: 96.2 ± 3.0 %; Table 2) and confirmed single amplicons via melt curve analysis.

**Table 2:** Primer sequences used in qPCR.

Gene (abbreviation)	NCBI accession number	Amplicon length (base pairs)	Efficiency (%)		Primer sequence
Beta actin (*ACTB*)	NM_205518.1	172	97.8	F	CTGACTGACCGCGTTACTCC
				R	CATACCAACCATCACACCCTGA
Brain-derived neurotropic factor (*BDNF*)	NM_001031616.1	147	92.8	F	TGAGACCAAATGCAACCCCA
				R	ATAAACCGCCAGCCAACTCT
Peptidylprolyl isomerase A (*PPIA*)	NM_001166326.1	122	98.1	F	GGGATTTGGCTACAAGGGCT
				R	CGGCAAACTTCTCCCCGTAA

Primers for each gene were designed on gene sequences specific to *Gallus gallus* (see NCBI Accession number) using the NCBI Primer-BLAST tool.

Primer efficiency was determined by running a standard curve consisting of five 10-fold serial dilutions of purified PCR product. All primers were designed to be exon-spanning.

We determined relative expression for *BDNF* using the ddCt method (Livak and Schmittgen, 2001). Briefly, *BDNF* expression for each sample was normalized to the geometric mean of *ACTB* and *PPIA (*dCt*),* then calculated relative to the reference treatment group (ddCt). Here, the FL rearing environment was used as a reference because those pullets did not have access to elevated space. We report relative expression as fold change (2^−ddCt^).

### Statistical Analysis

A completely randomized design with three treatments was used, where pen was the experimental unit. For statistical analysis of gene expression, R statistical software (version 4.0.4, R Core Team, 2021) was used. We evaluated the effect of rearing environment (factor with 3 levels: FL, ST, and TT) on mean reference gene expression (*ACTB, PPIA*) or fold change for the gene of interest (*BDNF*, where FL was the reference group) with linear mixed effect models (nlme package, version 3.1.152; Pinheiro et al., 2021), with pen as a random effect. Model assumptions were checked by visually evaluating the Q–Q plot, distribution of residuals, and homoscedasticity (sjPlot package, version 2.8.10, Lüdecke, 2021). Fold change values were log transformed to meet model assumptions.

Golgi-Cox statistical analyses were conducted in JMP Pro (version 16, SAS Institute, Cary, NC). For the Sholl analysis, AUC, and non-Sholl neuronal metrics, a mixed model was used with rearing environment (factor with 3 levels: FL, ST, and TT) and hippocampal subregion (factor with 3 levels: V, DM, and DL) as fixed effects. An interaction of rearing environment and hippocampal subregion was not significant, and its exclusion resulted in a lower AIC for all models, indicating a better model fit. For the Sholl analysis model, random effects included Sholl radius nested within neuron ID, neuron ID nested within pullet ID, and pullet ID nested within pen. The model also used a Repeated Covariance Structure with a first-order autoregression structure (AR(1)) that held pullet ID as the subject (described in Wilson et al., 2017). For AUC and non-Sholl neuronal metrics, a Residual structure was specified for the Repeated Covariance Structure, and pullet ID nested within pen was used as a random effect. AUC and non-Sholl neuronal metrics did not appear to be normally distributed based on histograms and residual plots, so they were natural log transformed to achieve normality. If a fixed effect was statistically significant, posthoc pairwise comparisons were made between levels using Tukey-Kramer HSD tests.

R statistical software (version 4.0.4, R Core Team) was also used to analyze correlations between individual pullet behavior on cognitive tasks and the individual's neural outcomes. A Spearman's rank correlation was utilized to evaluate the relationships between neural outcomes and continuous behaviors (ggpubr package, version 0.4.0; Kassambara, 2020). Continuous behaviors on the visual cliff at 30 and 90 cm were the number of times looking down and latency to cross the cliff, and continuous behavior in the Y-maze was latency to exit the maze. A Mann-Whitney U test (“wilcox.test” function in base R, stats package, version 0.4.0) was used to evaluate the relationships between neural outcomes and binary behaviors. Binary behaviors included crossing the visual cliff at 30 and 90 cm and choosing the short arm of the Y-maze. Overall, correlations between 8 behaviors and 7 neural metrics were performed for a total of 56 tests. A Bonferroni correction for multiple comparisons was applied to adjust the α level of significance to *P* = 0.0009.

The number of pullets used in each correlation analysis was determined by the individuals included for each brain metric and their performance on the cognition task. A total of 38 pullets were included in the Golgi-Cox analysis. To evaluate the relationship between the 6 non-Sholl neuronal metrics and behaviors on the visual cliff task, the number of times each individual looked down and whether the pullet crossed the visual cliff at 30 and 90 cm were evaluated for all 38 pullets. The relationship between non-Sholl neuronal metrics and the latency to cross the visual cliff was evaluated only for the pullets that crossed (N = 14 and N = 11 pullets at 30 and 90 cm, respectively). For the Y-maze task, non-Sholl neuronal metrics were correlated with the latency to exit the maze and the choice of the short arm when exiting only for pullets that exited the maze (N = 22 pullets).

A total of 30 pullets were included in the *BDNF* gene expression analysis. Due to errors in sample labeling, the log fold change of *BDNF* expression was not able to be successfully matched to the individual behavior for 4 pullets; therefore, *BDNF* was correlated with 26 pullets for the number of times looking down and whether the pullet crossed the visual cliff at 30 and 90 cm. Of those 26 pullets, the relationship between the log fold change of *BDNF* and latency to cross was analyzed for the 8 pullets that crossed the cliff. Latency to exit the Y-maze and the choice of the short arm when exiting were correlated with *BDNF* only for pullets that exited the maze (N = 15 pullets).

## RESULTS

### Dendritic Morphology

**Figure 3:**
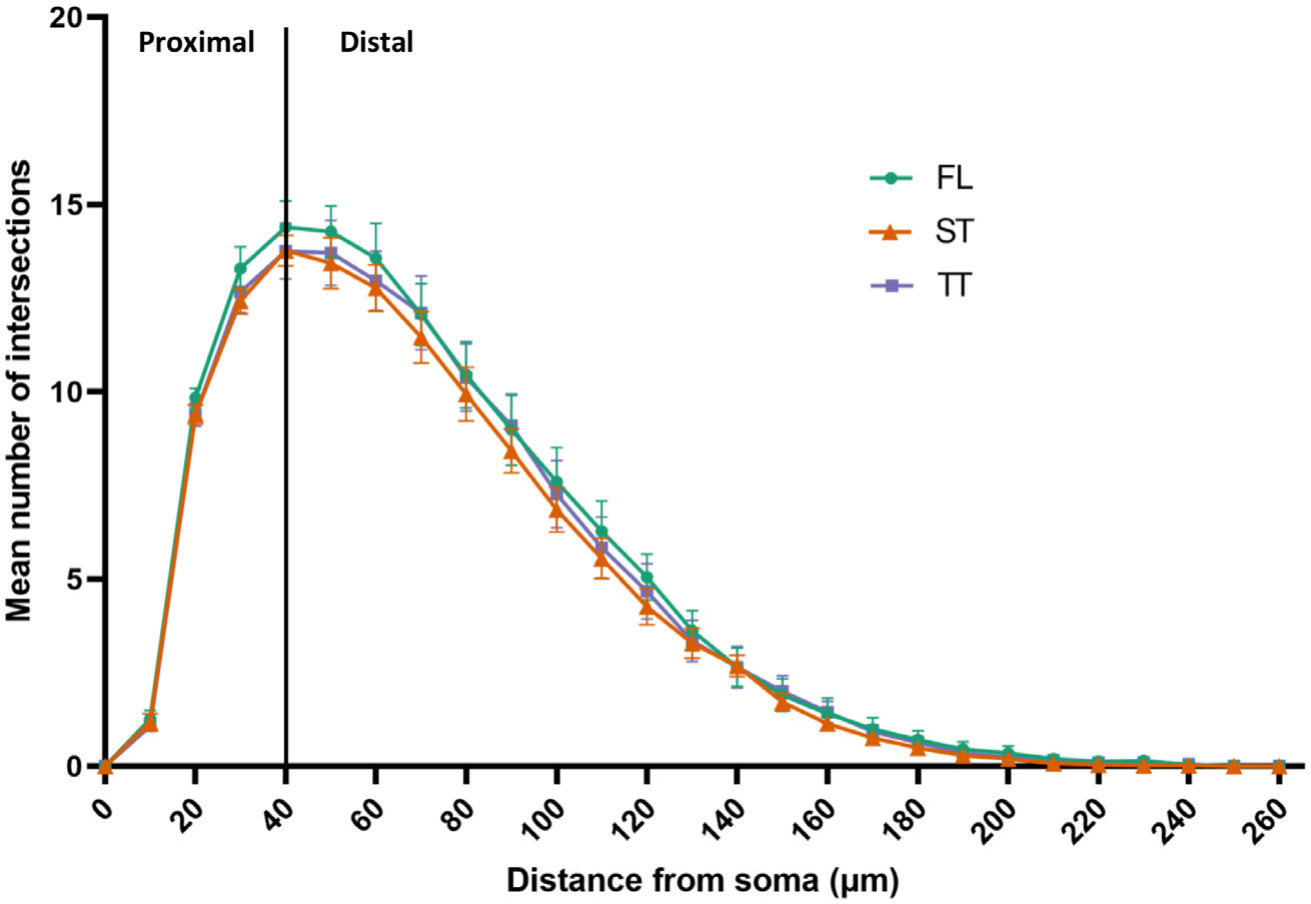
Sholl plots of the dendritic arbors from right hemisphere hippocampal multipolar neurons in female Dekalb White pullets at 16 wk of age. Pullets were reared on the floor (FL; n = 12 pullets, 51 total neurons), in a single-tiered aviary (ST; n = 13 pullets, 54 total neurons), or in a two-tiered aviary (TT; n = 13 pullets, 52 total neurons). Values are presented as mean ± SEM of the number of dendritic intersections at concentric rings in 10 μm segments from the soma (green circle for FL, orange triangle for ST, and purple square for TT). A black line at 40 μm represents the cutoff for proximal versus distal area under the curve analysis.

**Figure 4:**
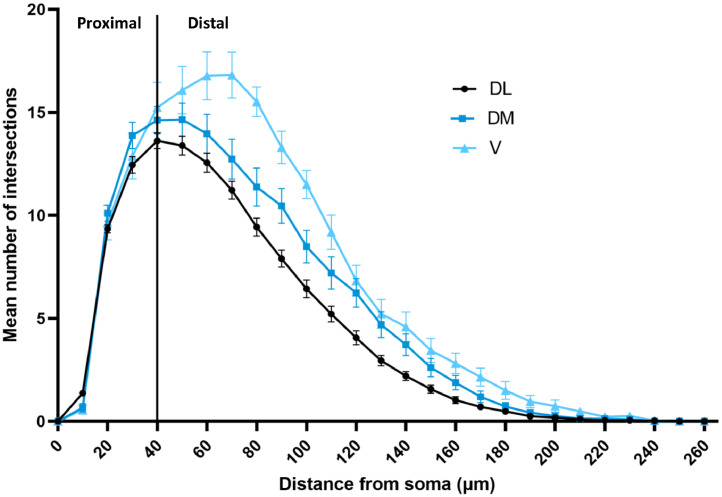
Sholl plots of the dendritic arbors from right hemisphere hippocampal multipolar neurons in female Dekalb White pullets at 16 wk of age. Neurons were sampled from hippocampal subregions dorsolateral (DL; n = 36 pullets, 109 total neurons), dorsomedial (n = 20 pullets, 35 total neurons), and ventral (V; n = 9 pullets, 13 total neurons). Values are presented as mean ± SEM of the number of dendritic intersections at concentric rings in 10 μm segments from the soma (black circle for DL, blue square for DM, and light blue triangle for V). A black line at 40 μm represents the cutoff for proximal versus distal area under the curve analysis.

The Sholl analysis indicated that rearing environment did not affect the dendritic arborization for multipolar hippocampal neurons (*F*_2,26_ = 0.069, *P* = 0.93; Figure 3), and no other morphological differences were noted between rearing environments (Table 3). However, the Sholl analysis showed that there are differences in dendritic arborization amongst subregions of the hippocampus (*F*_2,50_ = 4.05, *P* = 0.03; Figure 4). Specifically, V multipolar neurons have a higher number of intersections between dendrites and Sholl rings compared to DM and DL multipolar neurons overall and a higher AUC in the distal arbor than DL (Table 4). Other morphological differences were noted between the hippocampal subregions, such that V multipolar neurons also have longer dendrites on average compared to DM and DL multipolar neurons (*F*_2,144.9_ = 5.62, *P* = 0.005). V neurons have longer dendrites in sum (*F*_2,150.5_ = 7.20, *P* = 0.001), more terminal tips (*F*_2,138_ = 3.34, *P* = 0.04), and more branching nodes than DL neurons (*F*_2,135.2_ = 3.66, *p* = 0.03; Table 4). DL multipolar neurons have a smaller area of the soma compared to DM and V multipolar neurons (*F*_2,137.3_ = 9.94, *P* < 0.0001).

**Table 3:** Dendritic morphology parameters of hippocampal multipolar neurons from female Dekalb white pullets at 16 wk of age after being reared in different environments.

Morphology parameter	FL Estimate (95% CI)	ST Estimate (95% CI)	TT Estimate (95% CI)	*P*-value
Area under Sholl curve (AUC)	1,342.6 (1,184.3, 1,521.9)	1,329.2 (1,171.3, 1,508.4)	1,335.2 (1,180.0, 1510.9)	0.99
Proximal AUC (0–40 μm)	307.4 (288.8, 327.2)	298.4 (279.8, 318.2)	295.9 (278.1, 314.9)	0.59
Distal AUC (40–260 μm)	1,004.8 (854.0, 1182.3)	1,011.6 (858.5, 1192.0)	1,017.1 (866.4, 1,194.0)	0.99
Mean dendritic length (μm)	197.2 (174.8, 222.5)	207.9 (183.9, 235.0)	211.4 (187.6, 238.3)	0.64
Sum dendritic length (μm)	1,686.7 (1,488.9, 1,910.9)	1,668.8 (1,472.0, 1,892.0)	1,684.2 (1,489.5, 1,904.3)	0.99
Dendrites, *n*	8.5 (8.0, 9.0)	8.1 (7.6, 8.6)	8.0 (7.5, 8.5)	0.23
Terminal tips, *n*	26.6 (24.3, 29.2)	24.9 (22.6, 27.3)	26.2 (24.0, 28.7)	0.44
Branching nodes, *n*	17.3 (15.1, 19.8)	16.1 (14.0, 18.5)	16.7 (14.6, 19.1)	0.68
Soma size (μm^2^)	301.6 (282.1, 322.3)	306.5 (286.2, 328.2)	312.5 (292.5, 333.9)	0.69

Values were analyzed with a natural log transformation and are presented as back-transformed estimates for each rearing environment: floor (FL; n = 12 pullets, 51 total neurons), single-tiered aviary (ST; n = 13 pullets, 54 total neurons), and two-tiered aviary (TT; n = 13 pullets, 52 total neurons).

There were no significant differences for any morphology parameter across rearing environments (*P* > 0.05).

**Table 4:** Dendritic morphology parameters of hippocampal multipolar neurons from different hippocampal subregions from female Dekalb white pullets at 16 wk of age.

Morphology parameter	V Estimate (95% CI)	DM Estimate (95% CI)	DL Estimate (95% CI)	P-value
Area under Sholl curve (AUC)	1,671.0 (1,373.2, 2,033.5)^A^	1290.3 (1,140.3, 1,460.1)^ab^	1105.1 (1,026.3, 1,189.9)^b^	0.0002
Proximal AUC	304.3 (272.0, 340.4)	304.4 (284.3, 325.9)	293.0 (282.2, 304.2)	0.56
Distal AUC	1,358.6 (1,051.8, 1,754.8)^A^	963.0 (819.9, 1,131.2)^ab^	790.2 (717.8, 869.8)^b^	0.0002
Mean dendritic length (μm)	259.8 (212.9, 317.1)^A^	182.5 (161.2, 206.6)^b^	182.8 (170.1, 196.4)^b^	0.005
Sum dendritic length (μm)	2061.3 (1,701.5, 2,497.1)^A^	1607.9 (1,424.3, 1,815.3)^ab^	1430.4 (1,329.1, 1,539.3)^b^	0.001
Dendrites, n	8.1 (7.2, 9.0)	8.6 (8.1, 9.2)	7.9 (7.6, 8.1)	0.06
Tips, n	29.5 (25.2, 34.6)^A^	24.7 (22.4, 27.2)^ab^	23.8 (22.5, 25.1)^b^	0.04
Branching nodes, n	20.8 (16.5, 26.3)^A^	15.1 (13.0, 17.4)^ab^	14.9 (13.7, 16.1)^b^	0.03
Soma size (μm^2^)	331.5 (295.0, 372.5)^A^	318.4 (296.4, 342.0)^a^	273.6 (262.8, 284.7)^b^	<0.0001

Values were analyzed with a natural log transformation and are presented as back-transformed estimates from subregions within the hippocampus: ventral (V, n = 9 pullets, 13 total neurons), dorsomedial (DM, n = 20 pullets, 35 total neurons), and dorsolateral (DL, n = 36 pullets, 109 total neurons).

abAB Within a row, back-transformed estimates having different superscripts are significantly different (*P* < 0.05).

### BDNF Gene Expression

**Figure 5:**
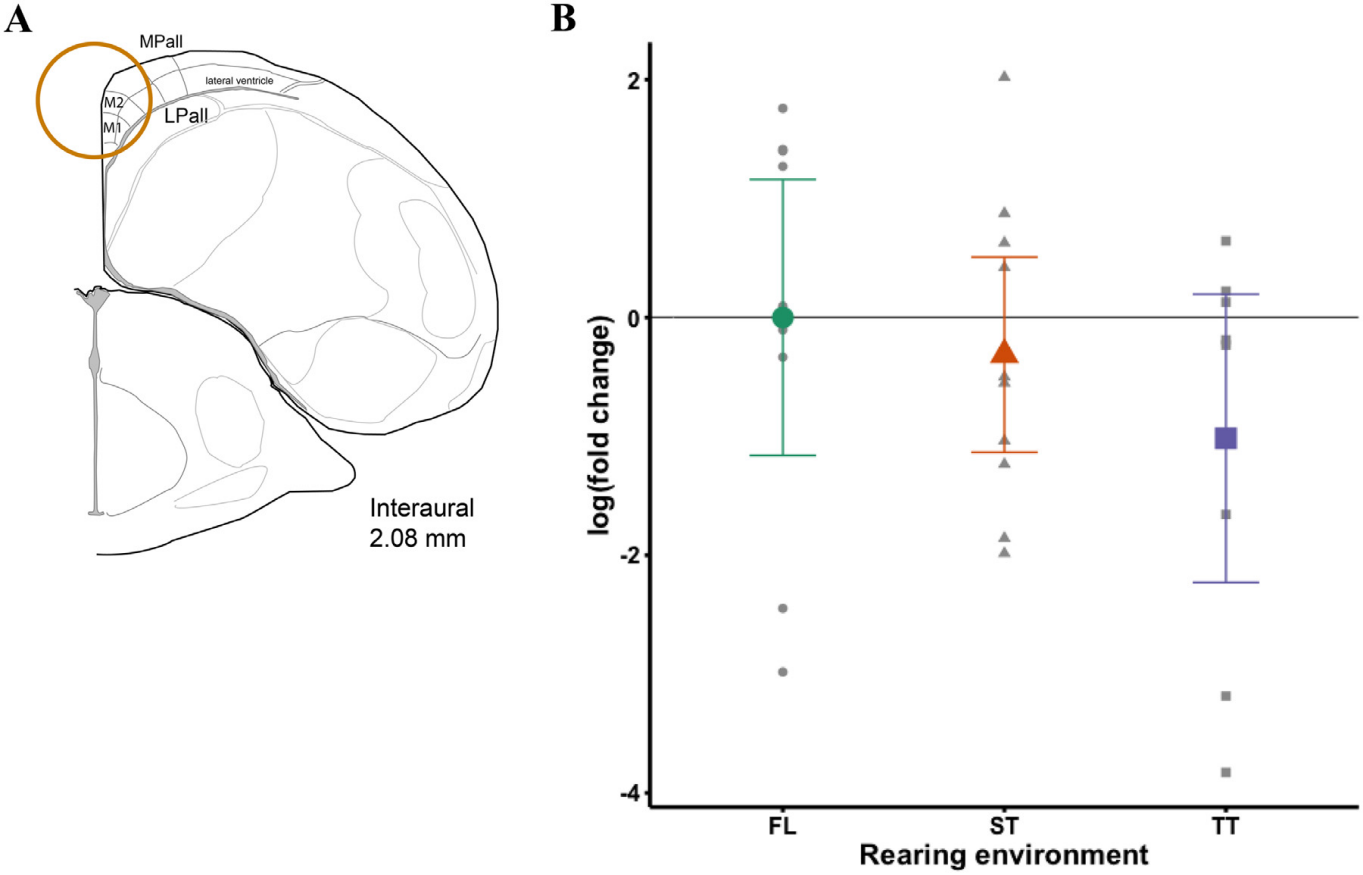
(A) Representative punch of hippocampal tissue taken from above the lateral ventricle in the medial, superior part of the left hemisphere. Figure recreated based upon the chick brain stereotaxic atlas (Puelles et al., 2007, Figure 20). (B) Log-transformed fold change of brain-derived neurotrophic factor (*BDNF*) gene expression in the hippocampus. Female Dekalb White pullets were reared on the floor (FL), in a single-tiered aviary (ST), or in a two-tiered aviary (TT) until 16 wk of age. Left hemisphere hippocampus was biopsied for real-time qPCR analysis of *BDNF* expression. Values are presented as mean ± 95% CI of the log-transformed fold change in colored shapes (green circle for FL, orange triangle for ST, and purple square for TT). FL served as the reference group because pullets in this rearing environment did not have access to elevated structures. Data points for individual animals are displayed in gray shapes corresponding to rearing environment.

Rearing environment did not have a significant effect on relative *BDNF* expression (*F*_2,11_ = 1.16, *P* = 0.35; Figure 5).

### Neural Outcomes Correlated With Individual Behavior

The effect of rearing treatment on the number of times looking down, latency to cross, and whether or not pullets crossed the visual cliff was complex as it involved interactions with other ages not included in the present study (see Jones, 2021 for a full summary). Rearing environment did not affect the latency to exit the Y-maze, and all birds chose the shorter arm in the Y-maze (see Jones, 2021). *BDNF* gene expression did not significantly correlate with any behavior on the visual cliff or Y-maze cognitive tasks (*P* > 0.05; Table 5). None of the 6 non-Sholl neuronal metrics significantly correlated with individual pullet behavior on either cognitive task (*P* > 0.05; Table 5).

**Table 5:** Spearman's rho correlation coefficients or Mann-Whitney W-statistics for pairwise comparisons between neural outcomes (*BDNF* expression or morphology parameters from Golgi-Cox staining) and behaviors on a visual cliff or Y-maze cognitive task.

Behavior	*BDNF* Expression	Mean dendritic length (μm)	Sum dendritic length (μm)	Dendrites (n)	Terminal tips (n)	Branching nodes (n)	Soma size (μm^2^)
Visual Cliff							
Looking down at 30 cm (n)	r_s_ = 0.42 N = 26 *P* = 0.03	r_s_ = −0.030 N = 38 *P* = 0.86	r_s_ = −0.0061 N = 38 *P* = 0.97	r_s_ = 0.082 N = 38 *P* =0.62	r_s_ = −0.029 N = 38 *P* = 0.86	r_s_ = 0.021 N = 38 *P* = 0.90	r_s_ = 0.034 N = 38 *P* = 0.84
Looking down at 90 cm (n)	r_s_ = 0.18 N = 26 *P* = 0.37	r_s_ = 0.28 N = 38 *P* = 0.09	r_s_ = 0.29 N = 38 *P* = 0.07	r_s_ = 0.051 N = 38 *P* = 0.76	r_s_ = 0.23 N = 38 *P* = 0.16	r_s_ = 0.26 N = 38 *P* = 0.11	r_s_ = −0.28 N = 38 *P* = 0.09
Crossing at 30 cm (yes/no)	W = 103 N = 26 *P* = 0.09	W = 173 N = 38 *P* = 0.76	W = 177 N = 38 *P* = 0.67	W = 164 N = 38 *P* = 0.98	W = 169.5 N = 38 *P* = 0.84	W = 176 N = 38 *P* = 0.69	W = 131 N = 38 *P* = 0.34
Crossing at 90 cm (yes/no)	W = 95 N = 26 *P* = 0.22	W = 137 N = 38 *P* = 0.84	W = 140 N = 38 *P* = 0.76	W = 111.5 N = 38 *P* = 0.52	W = 142 N = 38 *P* = 0.71	W = 153 N = 38 *P* = 0.45	W = 98 N = 38 *P* = 0.28
Latency to cross at 30 cm (s)	r_s_ = 0.24 N = 8 *P* = 0.58	r_s_ = 0.28 N = 14 *P* = 0.33	r_s_ = 0.36 N = 14 *P* = 0.21	r_s_ = −0.022 N = 14 *P* = 0.94	r_s_ = 0.40 N = 14 *P* = 0.16	r_s_ = 0.39 N = 14 *P* = 0.17	r_s_ = −0.35 N = 14 *P* = 0.22
Latency to cross at 90 cm (s)	r_s_ = −0.26 N = 8 *P* = 0.54	r_s_ = −0.15 N = 11 *P* = 0.65	r_s_ = −0.26 N = 11 *P* = 0.43	r_s_ = 0.032 N = 11 *P* = 0.93	r_s_ = −0.073 N = 11 *P* = 0.84	r_s_ = −0.046 N = 11 *P* = 0.89	r_s_ = 0.036 N = 11 *P* = 0.92
Y-maze							
Latency to exit maze (s)	r_s_ = 0.021 N = 15 *P* = 0.94	r_s_ = −0.29 N = 22 *P* = 0.20	r_s_ = 0.0096 N = 22 *P* = 0.97	r_s_ = 0.37 N = 22 *P* = 0.09	r_s_ = 0.014 N = 22 *P* = 0.95	r_s_ = −0.086 N = 22 p = 0.70	r_s_ = −0.10 N = 22 *P* = 0.65
Choice of short arm (yes/no)	W = 17 N = 15 *P* = 0.95	W = 37 N = 22 *P* = 0.97	W = 33 N = 22 *P* = 0.84	W = 42 N = 22 *P* = 0.64	W = 36 N = 22 *P* = 1	W = 32 N = 22 *P* = 0.77	W = 36 N = 22 *P* = 1

A Bonferroni correction for multiple corrections established the α level of significance at *P* = 0.0009.

Values are for 16-wk-old female Dekalb white pullets.

## DISCUSSION

The objectives of this study were 1) to determine whether manipulating height provided to pullets during the first 16 wk of life affected hippocampal development, and 2) to assess if hippocampal metrics correlated with individual behavior in spatial cognition tasks. We found that rearing environment did not affect dendritic morphology of multipolar neurons in the right hemisphere hippocampus, but morphology differed across hippocampal subregions. Similarly, providing height to pullets did not affect *BDNF* gene expression in the left hemisphere hippocampus. The hippocampal metrics measured in this study did not correlate with any of the behaviors associated with the cognitive tasks.

### Dendritic Morphology

In a previous study, chicks reared with visual barriers to their imprinting stimulus had longer dendrites and more dendritic spines in the right hemisphere hippocampus at 16 d of age than chicks reared without visual barriers (Freire and Cheng, 2004). Key methodological differences between the previous study and our study, including those related to chick socialization, space allowance, and timing of sample collection, may explain why we did not observe an effect of rearing environment on hippocampal dendritic morphology. Specifically, Freire and Cheng (2004) socially isolated chicks until 7 d of age, then paired chicks in groups of 2 until 16 d of age. Birds in the present study did not experience social isolation as they were housed in groups of 55 to 56 birds already at 1 d of age, then in groups of 45 birds between 8 and 16 wk of age. Social isolation is a known stressor for chicks and used as a model for the anxiety-depression continuum (Warnick et al., 2009). There is limited research on isolation-induced hippocampal changes in chicks, but multiple studies in mammals have shown that stressful social experiences early in life affects a variety of neurological measurements in multiple brain regions, including hippocampal dendritic morphology (reviewed by Davidson and Mcewen, 2012). Rearing chicks in larger social groups as we did may buffer any changes to hippocampal dendritic morphology that might have occurred from structural differences in the physical environment.

Space allowance is another methodological consideration. Freire and Cheng (2004) reared chicks in a cardboard box measuring 0.30 × 0.25 × 0.30 m (l × w × h; 0.075 m^2^ area) for the first 7 d, followed by a larger box from 8 to 16 d of age (0.55 × 0.40 × 0.60 m; 0.22 m^2^ area). Chicks in the present study were reared in a pen with an area 124 and 42 times larger than the first and second boxes used in the previous study, respectively (3.05 × 3.05 × 2.74 m; 9.30 m^2^ area). More space likely provided more opportunities for physical activity, and higher rates of physical activity in mammals are associated with changes in hippocampal dendritic morphology, cell proliferation, and cell survival (reviewed by van Praag et al., 2000). Therefore, the limited social interactions and presumably lower rates of physical activity in the previous study may have created different neural conditions that were more susceptible to morphological changes induced by environmental structures. Accordingly, the range of length of hippocampal multipolar dendrites was previously reported as 85 to 115 μm (Freire and Cheng, 2004), which is about half of the average dendrite length found in our study. These differences in dendrite length support the notion of rearing environment cultivating different neural conditions between the 2 studies. Variation in dendrite length may have also been related to tissue thickness, with the former study assessing dendrites from 200 μm sections compared to 100 μm sections utilized in the present study. Dendrites commonly become shorter with age, so it is unlikely that the difference in length is due to different sampling time points (16 d of age vs. 16 wk of age; Pannese, 2011).

Age at the time of analysis likely also influences whether morphological changes are detectable in the brain. The previous study analyzed dendritic morphology at 16 d of age, shortly after a known shift in brain lateralization (Vallortigara et al., 1997). We evaluated dendritic morphology at 16 wk of age to capture the end of the rearing period and just prior to sexual maturity. Brain maturation reportedly persists through 8 to 10 wk of age for chickens (Rogers, 1995), so we anticipated that morphological changes would still be detectable after maturation was complete. A recent study investigating the influence of early-life environment on neuronal morphogenesis also failed to find morphological changes at 21 wk of age. Those researchers evaluated pullets reared in groups of 145 birds, either in a floor pen with no enrichments or in a pen where visual, auditory, and physical enrichments were exchanged or moved every 2 or 3 d for the first 3 wk of life (Campbell et al., 2018). The size of the telencephalon and hippocampus were measured using cresyl violet staining at 21 wk of age, and there were no significant differences between the two rearing environments. Therefore, measuring morphology at earlier time points may be a future methodological consideration, pending the research question. It is worth noting that other studies found morphological differences in older hens. At 48 wk of age, hens that had spent 32 wk in different adult environments showed changes in hippocampal soma size and tyrosine hydroxylase innervation patterns (Patzke et al., 2009). At 52 wk of age, birds that were reared with a foster hen for the first 7 wk of life had a greater degree of lateralization in hippocampal soma size compared to birds reared without a foster hen (Nordquist et al., 2013). These studies reflect additional methodological considerations. Birds may need longer experiences with their environment to show morphological changes in the hippocampus, and social influences during rearing may have more of an effect on hippocampal morphology than physical structures.

Golgi-Cox methodology revealed differences in morphology between hippocampal subregions, where V multipolar neurons notably differed on most dendritic characteristics from one or both of the other subregions examined in this study. Dendritic characteristics differentiated by the three hippocampal subregions have not been previously reported, and this variation can inform future methodological considerations for quantifying dendritic morphology in multipolar neurons. However, the results should be interpreted with caution considering that a limitation of this study is a low representation of neurons in the V subregion. Across subregions, we found an average of 8 dendrites per multipolar neuron, which is comparable to the range of 4 to 6 dendrites per multipolar neuron described previously (Tömböl et al., 2000). Our study appears to be the first to report the Sholl profile, number of nodes, and number of tips for chicken hippocampal multipolar neurons. Previous studies using Nissl (Nordquist et al., 2013) or cresyl violet (Patzke et al., 2009) staining techniques to quantify soma size in the chicken hippocampus have distinguished between DM and V subregions, where DM cells were found to be larger than V cells. Our findings differed in that DM and V cells were statistically similar, but cells from both regions were larger than DL cells. The discrepancies between our study and others for soma size may reflect staining technique differences between studies. The soma area for multipolar neurons across subregions in our study aligns with the multipolar soma diameter previously reported for 28-day-old chicks using Golgi-Cox methods (e.g., Tömböl et al., 2000 reported 20-μm cell diameter, which is an estimated soma area of 314 μm^2^). However, the hippocampal soma area reported with Nissl and cresyl violet techniques appears to be half of this size (91.8–127.2 μm^2^ for neurons with cresyl violet in 48 wk old hens in Patzke et al., 2009; 130.8 to 169.3 μm^2^ for neurons with Nissl in 52 wk old hens in Nordquist et al., 2013). Distinguishing between neuron types is not possible with Nissl staining as it does not adequately label neuronal processes (Pilati et al., 2008), so the previously reported hippocampal soma area may be associated with non-multipolar neurons. Furthermore, research in rodents comparing Golgi-Cox stain to Nissl and cresyl violet observed the latter stains to underestimate soma size, a notable methodological consideration for future work (Pilati et al., 2008). Taken together, our findings highlight the importance of distinguishing between hippocampal subregions to account for morphological variation, and future work may provide insight regarding their functional differences homologous to the mammalian hippocampal subregions (Gupta et al., 2012; Atoji et al., 2016). Quantifying the rostro-caudal locations of hippocampal cells is another anatomical consideration in future studies. Region-specific differences in cell proliferation were previously reported for free range laying hens (Armstrong et al., 2020).

### BDNF Gene Expression

Environmental enrichment that promotes physical activity, learning, and memory has been linked with higher *BDNF* expression by providing more opportunities for social interactions and engaging with novel objects (van Praag et al., 2000). We theorized that varying the height of structures provided to pullets would increase load bearing exercise to access elevated structures, as well as foster novel learning through the cognitive and visual experience of accessing the height. However, rearing environment did not impact hippocampal *BDNF* gene expression, which may be due to methodological differences in providing sensorimotor stimulation between our study and earlier research. Previous studies were successful in modifying *BDNF* levels by giving animals larger enclosures, social groups, and changing or moving objects in the environment frequently when compared to animals experiencing social isolation, a barren environment, or both (van Praag et al., 2000; Rossi et al., 2006; Cao et al., 2017). The size of social groups and space allowance were kept constant across rearing environments in the present study, so there may be either a similar level of barrenness, or alternatively a similar level of complexity, perceived by birds across the rearing environments tested. Despite birds using elevated structures by the second wk of rearing (M. Makagon, unpublished data), the load bearing exercise and cognitive and visual experience of accessing different levels of height were not sufficient to trigger changes in hippocampal *BDNF*.

A recent study on pullet rearing evaluated environments where social groups and barrenness differed significantly by comparing birds reared in conventional cages (groups of approximately 25 birds/cage until 16 wk of age) or multitiered aviaries (groups of approximately 25 birds/cage for the first 4 wk of life, then a group of thousands of birds in an aviary until 16 wk of age; Tahamtani et al., 2016). Pullet rearing environment did not affect hippocampal tyrosine hydroxylase at 20 or 24 wk of age. Tyrosine hydroxylase is a rate-limiting enzyme in dopamine biosynthesis that could have a downstream effect on *BDNF*, as dopamine release can induce *BDNF* expression (Tahamtani et al., 2016). In combination with our study, this recent research suggests that the pullet rearing environment may have minimal effects on hippocampal *BDNF* and dopaminergic pathways regardless of social or physical features. Future research should investigate other pathways in the hippocampus (e.g., markers of neurogenesis, Armstrong et al., 2020) or alternative brain regions that have been understudied for experience-induced changes in chickens, such as the cerebellum, forebrain (e.g., hypothalamus), nucleus rotundus, or visual Wulst. Likewise, future research could evaluate neurological outcomes after birds have experienced a challenge. For example, pullets reared with access to perches had higher hypothalamic *BDNF* gene expression at 53 d of age compared to pullets reared on a litter floor without perches when they were exposed to an environmental challenge (Yan et al., 2020). Even though our work found similar neural metrics across rearing environments at the end of the rearing period, the brains may respond differently once they are challenged with a stressor, like being moved into the novel adult laying hen environment. Taking more neurological measurements after challenges and into the laying period are areas of future research consideration.

Furthermore, the physical structures in the pens remained unchanged and in the same location from 1 d of age. Commercial housing manufacturers promote management practices where height does change throughout the rearing period. For example, pullets are released from various aviary enclosures at different heights and different ages, or pullets are exposed to structures that gradually rise in height throughout the rearing period (Chore-Time, 2019; Big Dutchman, 2022; Vencomatic Group, 2022). The timing of exposure to staged increases in height may trigger neurological changes that stationary environments do not influence, which has not been studied to our knowledge and warrants further investigation.

### Neural Outcomes Correlated With Individual Behavior

Neural correlates to individual differences in behavior have been identified across a range of species for a variety of behaviors. In chickens, metrics of adult hippocampal neurogenesis were correlated to individual hen's outdoor range use (Armstrong et al., 2020). Previous work on pullet rearing did not correlate individual neural metrics with individual behavior, but at the group level, researchers found no relationships between hippocampal measurements and performance in spatial cognition tasks. For example, chickens reared in conventional cages took, on average, longer to complete a holeboard task and demonstrated poorer working memory during the reversal phase of the task than chickens reared in multitiered aviaries (Tahamtani et al., 2015). However, those same bird groups did not differ between rearing environments in staining intensity for tyrosine hydroxylase in the hippocampus and the caudolateral nidopallium (Tahamtani et al., 2016). Similarly, chickens reared with sensorimotor enrichment were, on average, faster to learn a T-maze task than chickens reared without enrichment, but there were no differences between groups for volume of the hippocampus or telencephalon (Campbell et al., 2018). In the present study, birds were subsampled from a different experiment as part of a larger project that demonstrated that rearing environment had complex effects on behavior in the visual cliff task (Jones, 2021). We theorized that dendritic morphology and *BDNF* in the hippocampus would correlate with individual differences in behavior, but we did not find significant relationships. Performance on the visual cliff and Y-maze tasks may have indicated different visuo-motor skills rather than skills involving spatial orientation, memory, and navigation. The former is associated with a different brain region (e.g., visual Wulst; Medina and Reiner, 2000).

Previous work in chickens has commonly evaluated 2 brain regions of interest with regard to spatial cognition, and the present study evaluated one region. Research in humans showed how interactions among a network of brain regions explained individual differences in spatial orientation ability (Arnold et al., 2014). Future studies investigating neural correlates for spatial behavior in individual birds should consider a similar network approach. For example, visual processing of impending collisions with looming objects involves the thalamofugal and tectofugal pathways in pigeons (Wu et al., 2005; Liu et al., 2008). These pathways may also be relevant in a network of processing spatial information in chickens, or different networks may be more significant for chickens considering that they are terrestrial and pigeons are arboreal in their spatial abilities. Finally, some individual differences in behavior may be strategies associated with personality (i.e., consistent interindividual variation in behavior; Dall et al., 2004). Future research aiming to explain individual differences in spatial behavior may consider neural correlated of coping styles (Armstrong et al., 2020), as well as correlations across multiple behaviors that categorize personality type, such as exploration, activity, social interactions, and fearfulness (reviewed in Garnham and Løvlie, 2018; Campbell et al., 2021).

In conclusion, modifying only height in the pullet rearing environment did not affect hippocampal dendritic morphology or hippocampal *BDNF*. Additionally, none of these hippocampal measurements correlated with individual differences in behavior on visual cliff or Y-maze tasks. Future work evaluating the relationship between pullet rearing environments and neural outcomes should consider measuring other pathways in the hippocampus (e.g., neurogenesis) or investigating a network of other brain regions. If the hippocampus is pursued further, the subregion should be identified as we found significant morphological differences for multipolar neurons amongst the subregion. The timing of when height or other novelty is introduced, the duration of exposure to the novelty, and possible social influences should also be considered when designing the rearing environment. Finally, there is much evidence that the rearing environment does modify bird behavior. If neural correlates are not able to be identified to explain the behavior changes, then other approaches to investigate the underlying cause are warranted (e.g., animal personality).
